# Healthcare Worker Attitudes and Perceptions toward Ebola Vaccine, United States, 2024

**DOI:** 10.3201/eid3112.251078

**Published:** 2025-12

**Authors:** Rinki Goswami, Anthony Lo Piccolo, Rachel Miller, Maria Frank, Corri Levine, Justin Chan

**Affiliations:** New York University Grossman School of Medicine, New York, New York, USA (R. Goswami, A. Lo Piccolo, J. Chan); NYC Health and Hospitals/Bellevue, New York (A. Lo Piccolo, J. Chan); Denver Health and Hospital Authority, Denver, Colorado, USA (R. Miller, M. Frank); University of Texas Medical Branch, Galveston, Texas, USA (C. Levine)

**Keywords:** Ebola virus disease, viruses, zoonoses, Zaire ebolavirus, *Orthoebolavirus zairense*, vaccines, occupational health, vaccine perceptions, United States

## Abstract

Our 2024 survey of eligible US healthcare workers found that 48% of unvaccinated healthcare workers are interested in receiving Ebola virus vaccine. The Advisory Committee on Immunization Practices recommended vaccination for healthcare workers at highest risk for occupational exposure to Zaire ebolavirus (*Orthoebolavirus zairense*). Addressing concerns identified by survey respondents might improve vaccine acceptance.

Orthoebolaviruses can be transmitted zoonotically or through exposure to bodily fluids from patients with Ebola disease (*1*). *Orthoebolavirus zairense* (Zaire ebolavirus) is the species most associated with outbreaks (*2*). During the 2014–2016 Zaire ebolavirus epidemic in West Africa, healthcare workers (HCWs) were infected and died from Ebola virus disease (EVD) at much higher rates than the general population (*3*). Four EVD cases were diagnosed in the United States, including 2 nurses involved in the care of an infected patient (*4*).

In 2019, the US Food and Drug Administration approved the Ebola vaccine ERVEBO (Merck, https://www.merck.com). The US Advisory Committee on Immunization Practices (ACIP) recommends the vaccine as a preexposure vaccination for persons at the highest risk of occupational exposure to Zaire ebolavirus (*5*,*6*). Eligible occupational risk groups include those responding to an outbreak, working as personnel at special pathogens treatment centers, or working as laboratorians handling specimens that might contain Zaire ebolavirus.

ERVEBO is highly effective in preventing disease caused by Zaire ebolavirus*.* The vaccine is a single-dose intramuscular injection that uses a live, attenuated recombinant vesicular stomatitis virus (VSV) to display the Ebola virus (strain Kikwit 1995) surface glycoprotein. An open-label, cluster-randomized ring vaccination trial done in Guinea and Sierra Leone estimated a vaccine efficacy of 100% (95% CI 79.3%–100.0%) (*7*). Subsequent observational studies also demonstrated vaccine effectiveness against Ebola virus transmission and death (*8*,*9*).

The real-world effectiveness of the vaccine in preventing EVD also depends on the willingness of HCWs to receive it. In this article, we describe the perceptions, attitudes, and desire to be vaccinated with ERVEBO among a sample of eligible HCWs from the United States.

## The Study

We conducted a cross-sectional online anonymous survey (Appendix) through REDCap (*10*) during March–October 2024. We distributed the survey to HCWs eligible for ERVEBO vaccination on the basis of ACIP guidelines (*5*,*6*) at 3 Regional Emerging Special Pathogen Treatment Centers (RESPTCs): NYC Health + Hospitals/Bellevue (New York, NY, USA); University of Texas Medical Branch (Galveston, TX, USA); and Denver Health and Hospital Authority (Denver, CO, USA) (*11*). We selected those survey sites because they were planning to offer ERVEBO vaccine to eligible staff. The study was approved by the New York University Grossman School of Medicine Institutional Review Board (approval no. i23-00730). We conducted recruitment through email communications (68%) and in-person invitations during optional vaccine education sessions (32%). The cohort we labeled as “interested in receiving vaccine” was made up of respondents who indicated they were already vaccinated or would definitely or probably choose to be vaccinated. The cohort we labeled as “not interested in receiving vaccine or unsure” was made up of respondents who indicated they would definitely or probably not choose to be vaccinated or were unsure. We compared characteristics of the 2 cohorts by using a χ^2^ test and considered a p value <0.05 significant.

There were 66 respondents (37% response rate). The largest age group was 30–49 years of age (64%); 63.6% were female and 36.4% male (Table 1). Occupations represented included physicians (42%), nurses (27%), clinical laboratory professionals (24%), emergency medical technicians (5%), and research laboratory professionals (2%). The most represented hospital departments were critical care medicine (27%), hospital medicine (11%), infectious diseases (11%), and pediatric critical care (11%). More than half of participants (56%) had received some form of education on Ebola vaccines, most commonly through informational sheets or pamphlets (33%) or self-study of primary literature or public health guidelines (20%).

**Table 1:** Characteristics of respondents to survey of healthcare worker attitudes and perceptions toward Ebola vaccine, United States, 2024

Characteristic	Total no. (%), n = 66	No. (%) interested in receiving vaccine, n = 34	No. (%) not interested in receiving vaccine or unsure, n = 32	p value*
Age				0.437
18–29	5 (7.6)	2 (40.0)	3 (60.0)	
30–39	22 (33.3)	12 (54.5)	10 (45.5)	
40–49	20 (30.3)	11 (55.0)	9 (45.0)	
50–59	16 (24.2)	9 (56.2)	7 (43.8)	
>60	3 (4.5)	0	3 (100.0)	
Sex				0.745
F	42 (63.6)	21 (50.0)	21 (50.0)	
M	24 (36.4)	13 (54.2)	11 (45.8)	
Profession				0.225
Clinical laboratory staff	16 (24.2)	5 (31.3)	11 (68.7)	
Research laboratory staff	1 (1.5)	0	1 (100.0)	
Nurse	18 (27.3)	12 (66.7)	6 (33.3)	
Physician	28 (42.4)	15 (53.6)	13 (46.4)	
Emergency medical technician	3 (4.5)	2 (66.7)	1 (33.3)	
Department				0.385
Emergency medicine	6 (9.1)	3 (50.0)	3 (50.0)	
Critical care medicine	18 (27.2)	11 (61.1)	7 (38.9)	
Hospital medicine	7 (10.6)	3 (42.9)	4 (57.1)	
Infectious diseases	7 (10.6)	5 (71.4)	2 (28.6)	
Pediatric critical care	7 (10.6)	5 (71.4)	2 (28.6)	
Other	1 (1.5)	0	1 (100.0)	
Unknown	20 (30.3)	7 (35.0)	13 (65.0)	
Have received Ebola vaccine education				0.463
Y	37 (56.1)	18 (48.6)	19 (51.4)	
N	28 (42.4)	16 (57.1)	12 (42.9)	
Prefer not to answer	1 (1.5)	0	1 (100.0)	

*By using χ^2^ test.

Thirty-four respondents (51%) were interested in receiving the vaccine, with a subset of respondents already vaccinated (n = 4) at the time of the survey (Figure, panel A). Among respondents interested and not already vaccinated, 27% would plan to get vaccinated when an EVD case appeared in the United States or their state or region (Figure, panel B).

**Figure:**
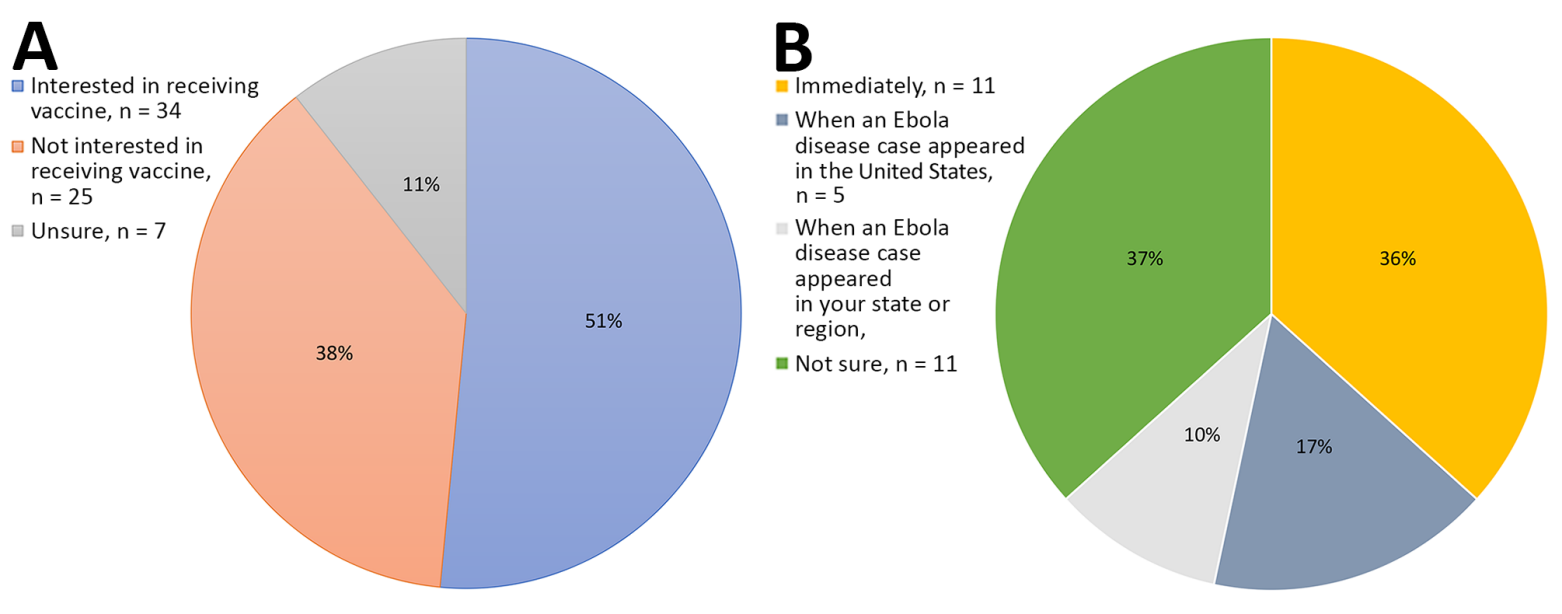
Survey responses from study of healthcare worker attitudes and perceptions toward the Ebola vaccine, United States, 2024. A) Interest in receiving ERVEBO vaccine (Merck, https://www.merck.com) among eligible healthcare workers at New York City Health + Hospitals/Bellevue (New York, NY, USA); University of Texas Medical Branch (Galveston, TX, USA); and Denver Health and Hospital Authority (Denver, CO, USA) (n = 66). B) Preferred timing for receiving ERVEBO among survey respondents who indicated they were interested in receiving vaccine and not already vaccinated (n = 30).

Among those respondents unsure about or not interested in receiving the vaccine (n = 32), some reported they might be convinced to vaccinate if there were an EVD outbreak in the United States (44%); if they better understood the risks and benefits of vaccination (34%), vaccine safety (31%), or the risk of spreading VSV to others (28%); and if there were an Ebola virus vaccine using a different vaccine technology (19%). In this cohort, the most common reasons for not wanting to receive ERVEBO vaccination or being unsure of their decision included concerns about spreading VSV to others (44%), feeling that the risks of vaccine side effects are not acceptable (31%), not personally knowing enough about the vaccine to make a decision (31%), and not thinking there will be an EVD outbreak in the United States (28%).

Among all respondents, the most common concerns regarding side effects were the potential for serious side effects that lasted a long time or interfered with daily life (62%), the potential increased risk of spreading VSV to others (47%), and the potential increased risk for arthritis (36%). Many respondents expressed desire to learn more about ERVEBO (Table 2). The most selected educational topics of interest included the likelihood and nature of side effects from vaccination (67%), the likelihood and severity of spreading VSV to others (59%), and what their individual risk of getting EVD was (26%).

**Table 2:** Topics that respondents to survey of healthcare worker attitudes and perceptions toward Ebola vaccine would like to learn more about regarding Ebola virus vaccination, United States, 2024*

Topic	Total no. (%), n = 66	No. (%) interested in receiving vaccine, n = 34	No. (%) not interested in receiving vaccine or unsure, n = 32	p value†
Likelihood and nature of side effects from vaccination	44 (66.7)	23 (67.6)	21 (65.6)	0.862
Likelihood and severity of spreading vaccine virus (vesicular stomatitis virus) to others	39 (59.1)	24 (70.6)	15 (46.9)	0.050
Whether Ebola virus experts or other respected peers are getting vaccinated	14 (21.2)	8 (23.5)	6 (18.8)	0.635
Individual risk of getting Ebola virus disease	17 (25.8)	6 (17.6)	11 (34.4)	0.120
Facts about Ebola virus disease, including infectiousness and risk of severe sickness	13 (19.7)	9 (26.5)	4 (12.5)	0.154
Other‡	5 (7.6)	2 (5.9)	3 (9.4)	0.592
None of the above	8 (12.1)	3 (8.8)	5 (15.6)	0.397

*Survey question: What additional knowledge or education about the Ebola virus vaccine, ERVEBO [Merck, https://www.merck.com], would you like to receive? (Select all that apply). †By using χ^2^ test. ‡Four of 5 respondents indicated “long-term research on this vaccine.”

In 2022, ACIP expanded their occupational exposure to include laboratory workers within the Laboratory Response Network and staff at SPTCs. They conducted a knowledge, attitudes, and practices survey in October 2020, during an EVD outbreak in the Democratic Republic of the Congo (*6*). Among SPTCs, 54% of respondents reported willingness to be vaccinated. Vaccine willingness increased to 81% if they were given the choice of vaccination timing. Those responses are consistent with our findings that many HCWs interested in ERVEBO vaccination would prefer to wait until an EVD case is imported to the United States. However, the SPTC survey did not report on specific areas of interest for more education or what factors might influence currently uninterested HCWs to get vaccinated in the future.

Surveys conducted in the Democratic Republic of the Congo (*12*) and Uganda (*13*) indicated a strong interest among HCWs to receive an Ebola vaccine. However, those data might not be generalizable to HCWs in the United States because the risk for exposure to Ebola virus is lower. A survey in 2015 during the height of the 2014–2016 Ebola outbreak in West Africa found that only 34% of the US population were interested in receiving an Ebola vaccine (*14*). This survey did not specifically assess HCW interest, and it was performed before the US Food and Drug Administration licensure of ERVEBO.

## Conclusions

During a period with no EVD outbreaks, 48% (30/62) of eligible unvaccinated HCWs surveyed at 3 US RESPTCs were interested in receiving the ERVEBO Ebola vaccine. Of those interested and not already vaccinated, 27% preferred to postpone vaccination until there is a case of EVD in the United States or their state or region. 

One limitation of this study is that only 3 RESPTCs were surveyed, so their attitudes might not be representative of all US HCWs eligible for ERVEBO vaccination. Second, the sample size is too small to analyze for differences between subgroups of respondents. However, we did achieve a 37% survey response rate from a cohort of workers who are most likely to provide ongoing direct care to patients with EVD that require care in the United States.

 Deployment of ERVEBO vaccine to eligible HCWs in the United States might be optimized by addressing respondent concerns. Those concerns include improving education on the risks and benefits of ERVEBO vaccination, vaccine safety, and risk of vaccine viral vector transmission.

Appendix Additional information about healthcare worker attitudes and perceptions toward Ebola vaccine, USA, 2024.
