# Supplementary material for: Healthcare Worker Attitudes and Perceptions toward Ebola Vaccine, United States, 2024
Source: Emerg Infect Dis. 2025 Dec;31(12):2320–3. doi: 10.3201/eid3112.251078 (PMC12782269; doi:10.3201/eid3112.251078)
Supplement: Appendix — Additional information about healthcare worker attitudes and perceptions toward Ebola vaccine, USA, 2024. [file 25-1078-Techapp-s1.pdf]

Article DOI: https://doi.org/10.3201/eid3112.251078

EID cannot ensure accessibility for supplementary materials supplied by authors. Readers who have difficulty accessing supplementary content should contact the authors for assistance.

Healthcare Worker Attitudes and Perceptions toward Ebola Vaccine, United States, 2024

Appendix

Questions asked for cross-sectional online anonymous survey distributed to healthcare workers eligible for Ebola vaccine at 3 Regional Emerging Special Pathogen Treatment Centers (RESPTCs): NYC Health + Hospitals/Bellevue, University of Texas Medical Branch, and Denver Health & Hospital Authority.

Demographics & Characteristics

1. What is your age?

• 18–29 years

• 30–39 years

• 40–49 years

• 50–59 years

• 60+ years

• Would not like to disclose

2. What is your sex?

• Male

• Female

• Would not like to disclose

3. Which institution are you affiliated with?

• New York City Health + Hospitals/Bellevue

• University of Texas Medical Branch

• Denver Health and Hospital Authority

• Would not like to disclose

4. What is your profession or role in your organization?

• Environmental services staff **Link to #10a**

• Clinical laboratory staff **Link to #10a**

• Research laboratory staff **Link to #10b

• Non-clinical administrative staff (please specify role) **Link to #5, #10a**

• Advanced Practice Provider (APP) (e.g., nurse practitioner or physician assistant) **Link to #5, 10.a**

• Nurse **Link to #5, 10.a**

• Physician **Link to #5, 10.a**

• Emergency medical technician (EMT) **Link to 10.a**

• Safety officer or trained observer **Link to 10.a**

• Respiratory Therapist **Link to 10.a**

• Other (please specify) **Link to 10a**

• Would not like to disclose **Link to 10a**

5. Which department do you work in?

• Emergency Medicine

• Critical Care Medicine

• Hospital Medicine

• Infectious Diseases

• Other (please specify)

• Would not like to disclose

Past Education

6. How informed do you feel about the Ebola virus vaccine, Ervebo?

• 5 = Extremely informed

• 4 = Moderately informed

• 3 = Somewhat informed

• 2 = Slightly informed

• 1 = Not at all informed

• I prefer not to answer this question

7. Have you received any form of previous education on Ebola vaccines?

• Yes **Link to #8**

• No

• I prefer not to answer this question

8. What form(s) of education have you previously received on the Ebola Virus vaccine, Ervebo? (select all that apply)

• In-person education session

• Attended a Webinar

• Informational sheets/pamphlets

• Self study of primary literature and/or public health guidelines

• Video presentation

• Organizational email blasts

• News Outlets/Social Media

• Discussing with medical staff

• Other (free text to describe)

• Would not like to disclose

Perception of Risk

9. How serious do you think getting infected with Ebola virus is (i.e., your impression of how severe the disease can be)?

• Very serious

• Serious

• Not serious

• I prefer not to answer this question

10a. How would you rate your risk of becoming infected with Ebola virus if a patient with Ebola virus disease went to your healthcare facility for care?

• High

• Intermediate

• Low

• Almost no risk

• I prefer not to answer this question

For research lab staff:

10b. How would you rate your risk of becoming infected with Ebola virus if handling specimens containing Ebola virus?

• High

• Intermediate

• Low

• Almost no risk

• I prefer not to answer this question

Vaccination status:

11. Have you already received the Ebola virus vaccine, Ervebo ?

• Yes **Link to #15, 18**

• No **Link to #12**

• Would not like to disclose **Link to #12**

Current Attitude Toward the Ebola Virus Vaccine, Ervebo

12. If you were eligible to receive the Ebola virus vaccine, Ervebo, would you choose to be vaccinated?

• Definitely yes **Link to #13,14, 18**

• Probably yes **Link to #13,14, 18**

• Probably no **Link to #16, #17, 18**

• Definitely no **Link to #16, #17, 18**

• Not sure **Link to #16, #17, 18**

• I prefer not to answer this question **Link to #18,#19 **

13. When would you choose to receive the Ebola virus vaccine, Ervebo?

• Immediately

• When an Ebola virus disease case appeared in the U.S.

• When an Ebola virus disease case appeared in your state/region

• Not sure

• I prefer not to answer this question

14. If you choose to get vaccinated or will likely get vaccinated, why? Select your top **3** reasons

• I feel the risks of side effects from this vaccine are acceptable.

• I feel the vaccine provides good protection against getting Ebola virus disease.

• I feel the vaccine provides good protection against dying from Ebola virus disease.

• I am concerned about the possibility of an Ebola outbreak in the United States

• I understand how this vaccine works and consider it a good vaccine option

• I understand the risk of spreading the vaccine virus (vesicular stomatitis virus, VSV) to others and consider it low

• I know I will not lose out on pay if I have to miss work due to side effects of receiving the vaccine

• I know my vaccination status will not impact who is expected to work with or near a patient with Ebola virus disease or their specimens

• I know Ebola virus experts or other peers I respect have gotten or will get vaccinated

• I understand Ebola virus disease causes severe sickness and high chance of death

• Other (please specify)

• I prefer not to answer this question

15. Why did you choose to get vaccinated? Select your top 3 reasons.

• I feel the risks of side effects from this vaccine are acceptable.

• I feel the vaccine provides good protection against getting Ebola virus disease.

• I feel the vaccine provides good protection against dying from Ebola virus disease.

• I am concerned about the possibility of an Ebola outbreak in the United States

• I understand how this vaccine works and consider it a good vaccine option

• I understand the risk of spreading the vaccine virus (vesicular stomatitis virus, VSV) to others and consider it low

• I know I will not lose out on pay if I have to miss work due to side effects of receiving the vaccine

• I know my vaccination status will not impact who is expected to work with or near a patient with Ebola virus disease or their specimens

• I know Ebola virus experts or other peers I respect have gotten or will get vaccinated

• I understand Ebola virus disease causes severe sickness and high chance of death

• Other (please specify)

• I prefer not to answer this question

16. If you would not want to receive the Ebola vaccine or are unsure, which of these reasons are most important to you? Select your top 3 reasons.

• I feel the risks of side effects from with this vaccine are **not** acceptable.

• I feel the vaccine does not provide good enough protection against getting Ebola virus disease.

• I feel the vaccine does not provide good enough protection against dying from Ebola virus disease.

• I don't think it is likely that there will be an Ebola virus outbreak in the United States

• I do not know enough about the vaccine to make a decision

• I am not comfortable with this type of vaccine technology that contains a live weakened virus (vesicular stomatitis virus, VSV)

• I am concerned I might spread the vaccine virus (vesicular stomatitis virus, VSV) to others, including family, friends, or patients

• I am concerned I may have to miss work after being vaccinated

• I am concerned I may lose pay or use up sick days after getting vaccinated if I am dealing with side effects

• If I get vaccinated, I am concerned I may be expected to work with or near a patient with Ebola virus disease or their specimens

• Many of my colleagues/peers are not planning on taking the vaccine

• I don't think I am medically eligible

• I don't think Ebola virus disease causes severe sickness and the chance of death is low

• Other (please specify)

• I prefer not to answer this question

17. If you are not interested in receiving the Ebola vaccine now or are unsure, are there factors that may convince you to accept it in the future? Select your top **3** reasons

• If I better understand the vaccine safety

• If I better understand the risks/benefits to being vaccinated

• If I better understand the effectiveness of the vaccine

• When there is an Ebola outbreak in the United States

• If there is an Ebola virus vaccine using a different vaccine technology, such as one that does not contain a live weakened virus (vesicular stomatitis virus, VSV)

• If I better understand the risks of spreading the vaccine virus (vesicular stomatitis virus, VSV) to others

• If I am reassured that if I needed to take time away from work due to side effects from the vaccine that I would not lose pay or sick days

• If I am reassured that Ebola vaccination status would not impact who is expected to work with or near a patient with Ebola virus disease or their specimens

• If I saw that Ebola virus experts or other peers I respect were getting vaccinated with the Ebola virus vaccine, Ervebo

• If I learned more about Ebola virus disease, such as how it spreads and the risk of serious sickness (please elaborate with specific questions in free text field)

• Other (please specify)

• None of the above

• I prefer not to answer this question

18. In deciding whether or not to be vaccinated, which of the following vaccine side effects are you **most** concerned about? (select all that apply)

• Potential for a serious side effect (for example, something that lasts for a long time or seriously interferes with my daily life)

• Potential increased risk of spread of the vaccine virus (vesicular stomatitis virus, VSV) to others, including family, friends, or patients

• Potential increased risk of arthritis (joint pain and inflammation)

• None

• Other (please specify)

• Pain, redness, or swelling at the injection site

- I prefer not to answer this question

Additional Knowledge

19. What additional knowledge or education about the Ebola virus vaccine, Ervebo, would you like to receive? (Select all that apply)

- Likelihood and nature of side effects from vaccination
- Likelihood and severity of spreading the vaccine virus (vesicular stomatitis virus, VSV) to others
- Whether Ebola virus experts or other peers I respect are getting vaccinated
- My individual risk of getting Ebola virus disease
- Facts about Ebola virus disease, including infectiousness and risk of serious sickness
- Other (please specify)
- None of the above
- I prefer not to answer this question
